# Systematic review of environmental factors associated with severe fever with thrombocytopenia syndrome occurrences

**DOI:** 10.1093/epirev/mxag011

**Published:** 2026-05-25

**Authors:** Gayoung Lee, Hyun-Kyung Kim, Arata Hidano, Sung-mok Jung

**Affiliations:** Department of Epidemiology, Gillings School of Global Public Health, University of North Carolina at Chapel Hill, Chapel Hill, NC, United States; Department of Public Health Sciences, Graduate School of Public Health, Seoul National University, Seoul, Republic of Korea; Department of Global Health and Development, Faculty of Public Health and Policy, London School of Hygiene and Tropical Medicine, London, United Kingdom; Department of Infectious Disease Epidemiology and Dynamics, Institute of Tropical Medicine, Nagasaki University, Nagasaki, Japan; Department of Infectious Disease Epidemiology and Dynamics, Institute of Tropical Medicine, Nagasaki University, Nagasaki, Japan; Saw Swee Hock School of Public Health, National University of Singapore, Singapore, Singapore; Carolina Population Center, University of North Carolina at Chapel Hill, Chapel Hill, NC, United States

**Keywords:** severe fever with thrombocytopenia syndrome, environmental impacts, One Health, systematic review

## Abstract

Severe fever with thrombocytopenia syndrome (SFTS) is an emerging tick-borne viral disease with a high fatality rate among older adults. It is posing a growing public health threat across Asia, with increasing potential for geographic spread beyond the region. Control efforts remain largely confined to individual-level tick-bite prevention, reflecting persistent gaps in understanding the ecological mechanisms that sustain SFTS transmission, particularly how environmental factors shape transmission dynamics across vectors, animal reservoirs, and human populations. We systematically reviewed 2910 studies to synthesize evidence on associations between environmental factors and SFTS occurrences across populations. Temperature, humidity, precipitation, elevation, and land cover are consistently linked to human SFTS occurrence through nonlinear, often reverse-U-shaped, relationships, underscoring the need for analytical frameworks capable of capturing the inherently nonlinear nature of environmental influences. However, we identified no quantitative assessments of how environmental factors shape SFTS occurrence in vectors or animal reservoirs, nor any stage-specific assessment of how these factors act across the transmission cycle, leaving multifaceted environmental effects acting across the tick–animal–human interface collapsed into oversimplified estimates based solely on human cases. To address these critical gaps, research must prioritize stage-specific elucidation of environmental drivers across the SFTS transmission cycle through mechanistic modeling approaches integrated with transboundary surveillance under a One Health framework. As climate and land-use changes continue to reshape vector habitats and expand regions at risk, such efforts will be essential for enhancing ecological understanding and guiding One Health–grounded surveillance and control strategies that can mitigate the growing transboundary burden of SFTS.

## Introduction

Severe fever with thrombocytopenia syndrome (SFTS) is an emerging tick-borne zoonotic disease caused by SFTS virus (SFTSV), posing a growing public health threat across Asia. First identified in China in 2010,^1^ the virus has since spread to neighboring countries, including Japan and the Republic of Korea (hereafter, South Korea), where sustained transmission to humans has been established. Clinically, SFTS manifests as high fever and thrombocytopenia and can progress to severe multiorgan failure. Age is a major factor of disease severity, with case fatality risks rising sharply from 4% to 9% in younger adults to 25% to 35% among those older than 60 years.^2^ Reflecting its substantial burden and the absence of accessible vaccines or antiviral treatments,^3^ the World Health Organization designated SFTS as 1 of 10 priority infectious diseases requiring urgent research.^4^

Despite such global recognition of its public health threat, effective control of SFTS remains challenging due to its complex ecological dynamics that sustain viral circulation and facilitate spillover to humans. *Haemaphysalis longicornis* has been identified as the primary tick vector (the virus has been occasionally detected in other tick species)^5^; however, its interactions with a wide range of reservoir hosts—including wild and domestic mammals and avian species^6^—remain poorly characterized. This uncertainty has impeded the development of ecologically grounded interventions, leaving current control strategies largely reliant on individual-level preventive measures (eg, tick bite avoidance such as wearing long sleeves during outdoor activities^7^), which are unlikely to effectively contain the continued geographic expansion of the virus.^8^ Although human SFTS cases are confined to Asia as of this writing, the detection of *H. longicornis* in the western Pacific islands^6^ and multiple US states^9^ has raised legitimate concerns about the potential emergence of new endemic areas.

**Figure 1:**
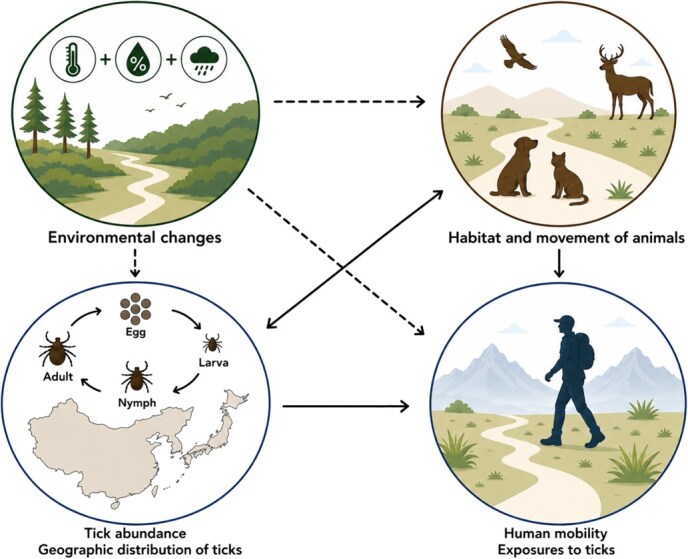
Schematic diagram of the influence of environmental factors on severe fever with thrombocytopenia syndrome transmission. Combined schematic representing the transmission dynamics of SFTSV (solid arrows) and the potential influence of environmental factors (dashed arrows) on tick vectors, animal, and human populations. The concept and scientific content of the figure were developed by the authors; ChatGPT (GPT-5.5; OpenAI) was used solely for graphical rendering.

In this context, synthesizing evidence on how environmental factors shape SFTS occurrence across vectors, animal hosts, and humans provides critical insights into its transmission dynamics within a broader ecological framework. Climatic and topographic conditions—including temperature, humidity, precipitation, and elevation—are well established as key determinants of tick-borne disease dynamics; they regulate the abundance, distribution, and behavior of both vectors and hosts (Figure 1).^10^ With accelerating global climate change, these drivers are expected to exert even greater influence, potentially reshaping vector habitats and facilitating the spread of pathogens into previously unaffected regions. However, the utility of such synthesis depends critically on the methodological rigor of individual studies. Existing studies often rely on coarse surveillance data and simplified analytical approaches that fail to capture the inherently dynamic nature (nonlinear) of ecological systems. These limitations may bias inferences about how environmental drivers influence transmission risk, particularly for emerging pathogens such as SFTSV, where ecological knowledge remains limited.

To address these challenges, we conducted a systematic review of the literature examining associations between environmental factors and SFTS occurrence across vectors, animal hosts, and human populations, with a focus on the analytic approaches used. This synthesis aims to consolidate current evidence and discuss key research priorities to inform more ecologically grounded surveillance and control strategies for SFTS.

## Methods

### Search strategy

**Figure 2:**
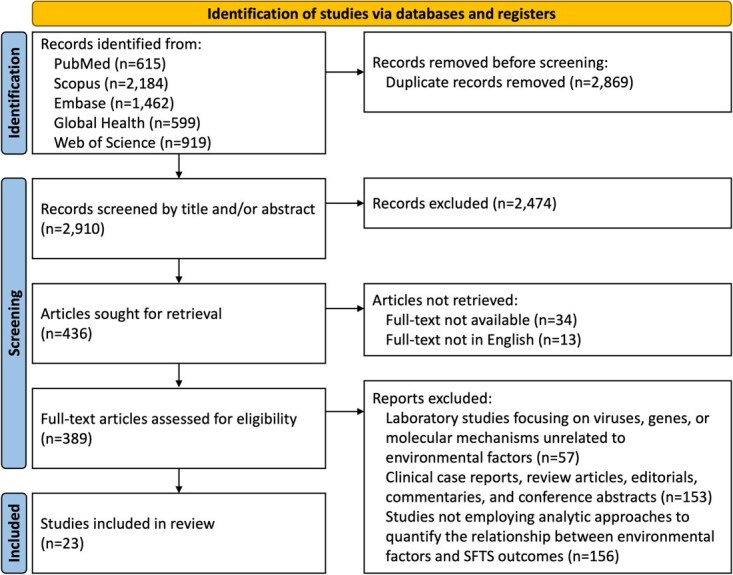
Preferred Reporting Items for Systematic Reviews and Meta-Analyses flow chart of study selection for a systematic review on severe fever with thrombocytopenia syndrome and environmental factors. SFTS, severe fever with thrombocytopenia syndrome.

We conducted a systematic review following the Preferred Reporting Items for Systematic Reviews and Meta-Analyses guidelines, with a prespecified protocol registered in the International Prospective Register of Systematic Reviews (Figure 2).^11^ Our search included articles published in English up to October 31, 2024, across 5 bibliographic databases: Embase, Global Health, PubMed, Scopus, and Web of Science. The search strategy combined terms for the key components: SFTS outcomes and environmental factors (including climatic and topographical variables). No restrictions were applied to specific epidemiologic outcomes, allowing for comprehensive inclusion of studies examining environmental influences on SFTS occurrence across vectors (ticks), nonhuman reservoirs (wildlife and domestic animals), and human populations. Search strings were adapted for the indexing syntax of each database; full details are provided in Table S1.

### Study selection

After removing duplicates, studies were screened for eligibility according to the following predefined criteria: (1) original studies using empirical SFTS outcome data (excluding those solely focused on tick distribution), and (2) studies using analytic approaches to quantify the relationship between environmental factors and SFTS outcomes beyond simple descriptions (excluding those only examining correlations). Further details on the eligibility criteria are provided in Table S2. Screening was conducted in 2 stages: an initial screening of titles and abstracts, followed by full-text review. Two authors independently performed the screening using Rayyan software. Any discrepancies were resolved through discussion and consensus, with adjudication by senior authors when necessary. To ensure comprehensive coverage, reference lists of included studies and relevant review articles were also manually screened.

### Quality assessment

Two authors independently assessed the risk of bias in all included studies, using the JBI Critical Appraisal Tool.^12^ This tool evaluates the risk of bias across 10 domains, with each domain rated as low (0), moderate (0.5), or high (1). Minor adaptations were made to address methodological nuances specific to this review, including the resolution of analyzed data and the suitability of applied analytic frameworks. Detailed descriptions of the modified assessment criteria and individual study assessments are provided in Table S3.

### Narrative synthesis

Data were extracted from the selected studies and systematically summarized in tables (Tables 1-3,^13-32^ and Tables S4-S9). Extracted information includes study period, geographic location (along with climate zones assigned based on the Trewartha classification^33^), environmental factors, SFTS outcomes, analytic methods, key findings, and time lag (defined as the time interval from environmental exposure to SFTS outcome) specifications. The key findings describe the nature of the relationships between environmental factors and SFTS outcomes. Specifically, where available, we extracted the range or category of environmental variables associated with the highest risk of SFTS outcomes—referred to as the “high-risk range” (or “high-risk point” when only a peak value was reported)—to facilitate interpretation of the overall shape of relationships (eg, skewness) along with the observed exposure ranges. When not explicitly provided in the primary reports, these values were approximated through visual inspection. Studies reporting only univariate results without any covariate adjustment were excluded from the narrative synthesis. All extracted data and interpretations were independently reviewed by all authors, with consensus reached to ensure the accuracy of the synthesis.

**Table 1:** Summary of included articles examining the association between temperature and severe fever with thrombocytopenia syndrome occurrence.

**First author (year)**	**Country**	**Region (climate zone** ^**a**^ **)**	**Study period**	**Model**	**SFTS outcome**	**Observational variables**	**Range of the main variable (min-max)**	**Relationship with the main variable** ^**b**^	**Lag specification**
Du (2014)^13^	China	Shandong (Temperate)	2010-2013	MaxEnt	Laboratory confirmed cases (timescale not reported)	Annual mean temperature	11.5-15°C¶	Exponential decay shape (high-risk range: 11.6-12.8°C)	Not stated
Yasuo (2019)^14^	Japan	Miyazaki (Subtropical)	2013-2018	GWLR	Annual laboratory confirmed cases	Annual mean temperature (2014)	Not reported	Direction not reported (not significant)^c^	Not stated
Miao (2020)^15^	China, Japan, South Korea	Nationwide	2010-2018	BRT coupled with MaxEnt	Laboratory confirmed cases (timescale not reported)	Annual mean temperature	−5 to -25°C¶	Reversed U -shape (high-risk range: 5-15°C)	Not stated
Sun (2021)^16^	China	Nationwide	2011-2018	ENM with MaxEnt	Annual laboratory confirmed cases	Annual mean temperature	−4 to 28°C	Reversed U shape (high-risk range: 12.5-17.5°C)	Not stated
Miao (2021)^17^	China	Nationwide	2010-2018	2-stage generalized BRT	Annual laboratory confirmed cases	Annual mean temperature	−3 to 26°C¶	Reversed U shape (high-risk range: 10-17°C)^d^	Not stated
Jiang (2022)^18^	China	Shandong (Temperate)	2010-2020	GAM	Cumulative laboratory confirmed cases across the study period	Annual mean temperature	12-16°C	Reversed U shape (high-risk point: 12.5-13°C)	Not stated
Nam (2023)^19^	South Korea	Nationwide	2013-2018	Bayesian regression model	Annual laboratory confirmed and probable cases	Annual mean max temperature	18.1-18.9°C	Negative (significant)^c^	Not stated
Tao (2024)^20^	China	Zhejiang (Subtropical)	2011-2019	MaxEnt	Laboratory confirmed and probable cases (timescale not reported)	Annual mean temperature	16.9-19.6°C	Declining shape (high-risk range: 16.9-17.8°C)	Not stated
Liu (2015)^21^	China	Nationwide	2010-2013	BRT coupled with MaxEnt	Monthly laboratory confirmed cases	Monthly mean temperature	5-27°C¶	Reversed U shape (high-risk range: 20-22°C)^d^	Lag examined, but not explictly reported
Wu (2020)^22^	China	Zhejiang (Subtropical)	2011-2018	Random forest model	Monthly laboratory confirmed cases	Monthly mean temperature	3-30°C (mean: 18.2°C)	Increasing shape with 2 peaks (high-risk range: 21.5-23.6°C, 28.9-30.0°C)	Not stated
Wang (2022)^23^	China	Liaoning (Temperate)	2011-2019	GLM	Monthly laboratory confirmed cases	Monthly mean temperature	Not reported (mean: 24.8°C)	Positive (significant) (0-5–week time lag)^c^	Lagged (1-5 weeks) and nonlagged effects examined
Ding (2023)^24^	China	Nationwide	2010-2019	GAM	Monthly laboratory confirmed cases	Monthly mean temperature	−10 to 30°C¶	Increasing shape (high-risk range: not defined)	Not stated
Ogawa (2024)^25^	Japan	Nationwide	2010–2021	Mixed–effects modified Poisson regression model	Monthly hospitalizations	Monthly mean temperature	Low: −1.1 to 11.6 °C (mean: 8°C) Middle: 11.7-15.0 °C (mean: 13.7°C) High: 15.1-23.9 °C (mean: 16.4°C)	Increasing shape^e^	Not stated
Sun (2018a)^26^	China	Henan (Temperate) Hubei, Anhui (Subtropical)	2011-2016	Quasi-Poisson GAM	Monthly laboratory confirmed cases	Monthly max temperature	5.1-35.2°C (median: 23.7°C)	Increasing shape (high-risk range: not defined)	Not stated
Zhang (2019)^27^	China	Jiangsu (Subtropical)	2010-2016	MaxEnt	Laboratory confirmed cases (timescale not reported)	Maximum temperature of warmest month	28-34.2°C	Reversed U shape (high-risk range: 32.8-34.2°C)	Not stated
Sun (2018b)^28^	China	Henan (Temperate) Hubei, Anhui (Subtropical)	2011-2015	DLNM	Weekly laboratory confirmed cases	Weekly mean temperature	−1.4 to 32.8°C (median: 17.7°C)	Reversed U shape (high-risk point: 23°C)	Cumulative lag effect (0-27 weeks) examined
Wang (2024)^29^	China	Jiaodong (Subtropical)	2014-2020	Quasi-Poisson GAM	Weekly laboratory confirmed cases	Weekly min temperature	−9.5 to 27.1°C (mean: 10.3°C)	Reversed U shape (high-risk point: 15-20°C)^d^	Cumulative lag effect (0-27 weeks) examined
Deng (2022)^30^	China	Jiangsu (Subtropical)	2017-2020	GAM	Daily laboratory confirmed cases	Daily mean temperature	−3.5 to 34°C (mean: 16.3°C)	Consistent risk followed by a sharp decline at 26°C (no time lag)	Lagged (30 days) and nonlagged effects examined
Wu (2016)^31^	China	Nationwide	2010-2012	GWLR	Binary detection status based on laboratory confirmed cases (timescale not reported)	Mean temperature from April to July across 2010-2012	Not reported	Positive (significant)^c^	Not stated
Wang (2017)^32^	China	Hubei (Subtropical)	2011-2016	Panel Poisson regression model	Annual laboratory confirmed and probable cases	Mean temperature of the 4 highest-incidence months each year	Not reported	Negative (significant)^c^	Not stated

Abbreviations: BRT, boosted regression tree; DLNM, distributed lag nonlinear model; ENM, ecological niche model; GAM, generalized additive model; GLM, generalized linear model; GWLR, geographically weighted logistic regression; Max, maximum; MaxEnt, maximum entropy; Min, minimum.

a Climate zone was classified following the Trewartha climate classification.

b High-risk range is defined as either (1) conditions under which the maximum entropy model estimated a severe fever with thrombocytopenia syndrome (SFTS) occurrence probability >50% or (2) the peak range reported by other models. When a range was not reported, the peak value is marked as the high-risk point.

c A linear relationship between SFTS outcome and environmental variables was assumed.

d Range was defined by the authors through visual inspection of the original figures.

e Shape defined by the relationship of risk measures between categories of outcome variables, rather than in an exposure-response curve.

**Table 2:** Summary of included articles examining the association between humidity and severe fever with thrombocytopenia syndrome occurrence.

**First author (year)**	**Country**	**Region (climate zone** ^**a**^ **)**	**Study period**	**Model**	**SFTS outcome**	**Observational variables**	**Range of the main variable (min-max)**	**Relationship with the main variable** ^**b**^	**Lag specification**
Sun (2021)^16^	China	Nationwide	2011-2018	ENM with MaxEnt	Annual laboratory confirmed cases	Annual mean relative humidity	24.3-91.4%	Reversed U shape (high-risk range: 63%-82%)	Not stated
Nam (2023)^19^	South Korea	Nationwide	2013-2018	Bayesian regression model	Annual laboratory confirmed and probable cases	Annual mean relative humidity	66.1%-68.8%	Negative (not significant)^c^	Not stated
Liu (2015)^21^	China	Nationwide	2010-2013	BRT coupled with MaxEnt	Monthly laboratory confirmed cases	Monthly mean relative humidity	30%-85%^d^	Reversed U shape (high-risk range 72%-75%)^d^	Lag examined, but not explicitly reported
Sun (2018a)^26^	China	Henan (Temperate) Hubei, Anhui (Subtropical)	2011-2016	Quasi-Poisson GAM	Monthly laboratory confirmed cases	Monthly mean relative humidity	51.9%-86.6% (median: 72.5%)	Increasing shape (high-risk range: not defined)	Not stated
Wu (2020)^22^	China	Zhejiang (Subtropical)	2011-2018	Random forest model	Monthly laboratory confirmed cases	Monthly mean relative humidity	60%-89% (mean: 75.4%)	Increasing shape (high-risk range: 81.4%-89%)	Not stated
Wang (2022)^23^	China	Liaoning (Temperate)	2011-2019	GLM	Monthly laboratory confirmed cases	Monthly mean relative humidity	Not reported (mean: 77.4%)	Positive (significant) (0-5–week time lag)^c^	Lagged (1-5 weeks) and nonlagged effects examined
Ding (2023)^24^	China	Nationwide	2010-2019	GAM	Monthly laboratory confirmed cases	Monthly mean relative humidity	30%-100%^d^	U-shape followed by plateau (high-risk range: 50%-100%)^d^	Not stated
Wang (2024)^29^	China	Jiaodong (Subtropical)	2014-2020	Quasi-Poisson GAM	Weekly laboratory confirmed cases	Weekly mean relative humidity	34.8%-87.6% (mean: 66.6%)	Increasing shape (high-risk range: not defined)	Cumulative lag effect (0-27 weeks) examined
Deng (2022)^30^	China	Jiangsu (Subtropical)	2017-2020	GAM	Estimated tick-to-human transmissibility	Daily mean relative humidity	31%-98%	Decreasing shape (30-day time lag)	Lagged (30 days) and nonlagged effects examined
Wu (2016)^31^	China	Nationwide	2010-2012	GWLR	Binary detection status based on laboratory confirmed cases (timescale not reported)	Mean relative humidity from April to July across 2010-2012	Not reported	Positive (significant)^c^	Not stated
Wang (2017)^32^	China	Hubei (Subtropical)	2011-2016	Panel Poisson regression model	Annual laboratory confirmed and probable cases	Mean relative humidity of the 4 highest-incidence months each year	Not reported	Positive (significant)^c^	Not stated

Abbreviations: BRT, boosted regression tree; ENM, ecological niche model; GAM, generalized additive model; GLM, generalized linear model; GWLR, geographically weighted logistic regression; MaxEnt, maximum entropy.

a Climate zone was classified following the Trewartha climate classification.

b High-risk range is defined as either (1) conditions under which the maximum entropy model estimated a severe fever with thrombocytopenia syndrome occurrence (SFTS) occurrence probability >50% or (2) the peak range reported by other models. When a range was not reported, the peak value is marked as the high-risk point.

c A linear relationship between SFTS outcome and environmental variables was assumed.

d Range was defined by the authors through visual inspection of the original figures.

**Table 3:** Summary of included papers examining the association between precipitation and SFTS occurrence.

**First author (year)**	**Country**	**Region (climate zone** ^**a**^ **)**	**Study period**	**Model**	**SFTS outcome**	**Observational variables**	**Range of the main variable, mm (min-max)**	**Relationship with the main variable** ^**b**^	**Lag specification**
Miao (2021)^17^	China	Nationwide	2010-2018	2-stage generalized BRT	Annual laboratory confirmed cases	Annual cumulative precipitation in driest quarter	0-420^c^	Increasing shape with a plateau at 80 mm^c^	Not stated
Sun (2021)^16^	China	Nationwide	2011–2018	ENM with MaxEnt	Annual laboratory confirmed cases	Annual cumulative precipitation	286.5-2244.9	Reversed U shape (high-risk range: 700-2250 mm)	Not stated
Duan (2023)^34^	China	Shandong (Temperate)	2010-2021	MaxEnt	Laboratory confirmed cases (timescale not reported)	Annual cumulative precipitation	400-1500^c^	Reversed U shape (high-risk range: 743.9-956.1 mm)	Not stated
Nam (2023)^19^	South Korea	Nationwide	2013-2018	Bayesian regression model	Annual laboratory confirmed and probable cases	Annual cumulative precipitation	934-1402	Positive (not significant)^d^	Not stated
Liu (2015)^21^	China	Nationwide	2010-2013	BRT coupled with MaxEnt	Monthly laboratory confirmed cases	Monthly cumulative precipitation	0-1750	Stepwise increasing shape (inflection at 600 mm)^c^	Lag examined, but not explicitly reported
Ogawa (2024)^25^	Japan	Nationwide	2010-2021	Mixed-effects modified Poisson regression model	Monthly hospitalizations	Monthly cumulative precipitation	Low: 675-1405 (mean: 1217) Middle: 1406-1851 (mean: 1608) High: 1852-3946 (mean: 2331)	U shape (not significant)^e^	Not stated
Wu (2020)^22^	China	Zhejiang (Subtropical)	2011-2018	Random forest model	Monthly laboratory confirmed cases	Monthly cumulative precipitation	16-326 (mean: 138.9)	Bimodal shape (high-risk range: 71.9-90.4 mm, 189.4-232.7 mm)	Not stated
Jiang (2022)^18^	China	Shandong (Temperate)	2010-2020	GAM	Total number of laboratory confirmed cases across the study period	Annual mean precipitation	400-750	Reversed U shape (high-risk point: 650 mm)	Not stated
Du (2014)^13^	China	Shandong (Temperate)	2010-2013	MaxEnt	Laboratory confirmed cases (timescale not reported)	January mean precipitation, Annual mean precipitation	2-12 (January mean precipitation)^c^	January mean precipitation: reversed U shape (high-risk range: 5.5-7.8 mm), annual mean precipitation: not reported (high-risk range: 640-660 mm)	Not stated
Wu (2016)^31^	China	Nationwide	2010-2012	GWLR	Binary detection status based on laboratory confirmed cases (timescale not reported)	Mean precipitation from April to July across 2010-2012	Not reported	Negative (significant)^d^	Not stated
Wang (2022)^23^	China	Liaoning (Temperate)	2011-2019	GLM	Monthly laboratory confirmed cases	Monthly mean precipitation	Not reported	Positive (significant) (0-5–week time lag)^d^	Lagged (1-5 weeks) and non-lagged effects examined
Ding (2023)^24^	China	Nationwide	2010-2019	GAM	Monthly laboratory confirmed cases	Monthly mean precipitation	0-1000^c^	Constant (high-risk range: not defined)	Not stated
Miao (2020)^15^	China, South Korea, and Japan	Nationwide	2010-2018	BRT coupled with MaxEnt	Laboratory confirmed cases (timescale not reported)	Cumulative precipitation of warmest quarter, cumulative precipitation of coldest quarter	10-100 (warmest quarter)^c^; 0-60 (coldest quarter)^c^	Logistic shape (warmest quarter) (high-risk range: 60-100 mm)^c^ Reversed U shape (coldest quarter) (high-risk range: 5-20 mm)^c^	Not stated
Tao (2024)^20^	China	Zhejiang (Subtropical)	2011-2019	MaxEnt	Laboratory confirmed and probable cases (timescale not reported)	Cumulative precipitation of wettest month	131.9-351.9	Declining shape (high-risk range: 131.9-224.4 mm)	Not stated

Abbreviations: BRT, boosted regression tree; ENM, ecological niche model; GAM, generalized additive model; GLM, generalized linear model; GWLR, geographically weighted logistic regression; MaxEnt, maximum entropy.

a Climate zone was classified following the Trewartha climate classification.

b High-risk range is defined as either (1) conditions under which the maximum entropy model estimated a severe fever with thrombocytopenia syndrome occurrence (SFTS) occurrence probability >50% or (2) the peak range reported by other models. When a range was not reported, the peak value is marked as the high-risk point.

c Range was defined by the authors through visual inspection of the original figures.

d A linear relationship between SFTS outcome and environmental variables was assumed.

e Shape defined by the relationship of risk measures between categories of outcome variables, rather than in an exposure-response curve.

## Results

### Summary of the included articles for review

**Figure 3:**
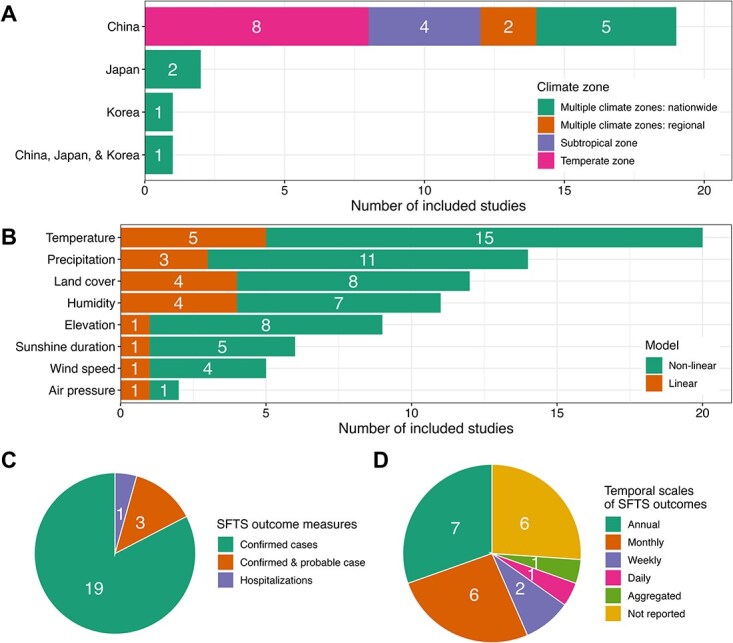
Summary of studies included in a systematic review on severe fever with thrombocytopenia syndrome (SFTS) and environmental factors. A) Number of studies by country and by the climate zone where data were collected. *Multiple climate zones: nationwide* denotes studies conducted at the national level in countries spanning several climate zones under the Trewartha classification, whereas *Multiple climate zones: regional* refers to province-level studies that covered multiple areas across more than 1 climate zone. B) Number of studies by environmental factors assessed, with classification based on whether linearity was assumed in the analytic approach. C) Proportion of studies by SFTS outcome measures, categorized as laboratory-confirmed cases only, confirmed and probable cases, or hospitalizations. D) Proportion of studies by temporal scales of SFTS outcomes. *Aggregated* refers to studies that applied binary indicators of case occurrence over a defined period longer than 1 year.

After screening 2910 articles, a total of 23 were included in the final synthesis to examine the associations between environmental factors and SFTS occurrence (Figure 3). The majority (*n* = 22 of 23 articles) analyzed data from a single country, predominantly China (*n* = 19), followed by Japan (*n* = 2) and South Korea (*n* = 1). One multicountry study covered China, Japan, and South Korea.^15^ Of the single-country studies, 15 were conducted at the subnational levels, mainly in historically SFTS-endemic regions (eg, Shandong, Hubei, Zhejiang).

Although our search strategy was designed to capture studies investigating SFTS occurrence across species (ie, tick vectors, nonhuman reservoirs, and humans), all studies included focused exclusively on human outcomes. In measuring human SFTS occurrence, all but 4 studies used laboratory-confirmed cases. Of the exceptions, 3 included both laboratory-confirmed and probable cases (ie, clinically diagnosed cases without laboratory confirmation),^19^^,^^20^^,^^32^ and 1 analyzed SFTS hospitalizations.^25^ Outcomes of SFTS were reported at varying temporal resolutions, ranging from annual to daily; however, 6 studies did not specify this information, which limited interpretability and comparability across studies.

Temperature was the most frequently examined environmental factor (*n* = 20), followed by precipitation (*n* = 14), land cover (*n* = 12), humidity (*n* = 11), and elevation (*n* = 9). Other environmental factors explored included sunshine duration (*n* = 6), wind speed (*n* = 5), and air pressure (*n* = 2). Environmental data were most aggregated at either the annual (*n* = 9) or monthly (*n* = 9) level, with each count indicating that at least 1 variable in the respective studies was measured on that scale. Other studies used weekly or daily scales, or specific summary metrics (eg, maximum temperature of the warmest month in each year). A detailed summary of environmental variables and their corresponding timescales in each study is provided in Table S4.

Most studies (*n* = 16) used statistical models that allow for potential nonlinearity in the relationship between environmental factors and SFTS outcomes, whereas a smaller subset (*n* = 7) applied models constraining the relationship to be linear.^14^^,^^19^^,^^23^^,^^31^^,^^32^^,^^35^^,^^36^ Among the nonlinear approaches, maximum entropy models were the most frequently used (*n* = 7), followed by generalized additive models (*n* = 5). Time lags were addressed in 5 studies (Table S4). Of these, 2 studies incorporated lag effects within the analytical framework by applying distributed lag models to estimate cumulative effects over the specified lag periods.^27^^,^^28^ The remaining 3 studies examined lag structures through variable definitions, for instance, by including lagged exposures (eg, temperature with a 1-month lag) as covariates^13^^,^^14^; however, 1 of these did not explicitly report how lagged effects were handled.^18^

### Temperature

A total of 20 studies examined the relationship between temperature and human SFTS outcomes (Table 1). Of these, 15 accounted for potential nonlinearity in the relationship: 8 reported a reverse-U-shaped relationship (defined as a pattern in which risk increases up to an optimal level followed by a subsequent decline),^15-18^^,^^21^^,^^27-29^ 4 observed an increasing trend,^22^^,^^24-26^ 2 identified a decreasing trend,^13^^,^^20^ and 1 reported a pattern of consistent risk followed by a sharp decline at higher temperature.^30^ Among studies reporting a reverse-U-shape, the high-risk range varied depending on both temporal (annual vs monthly) and spatial (nationwide vs subnational) measurement scales. Nationwide studies using annual mean temperatures typically identified lower high-risk ranges (approximately 12-15°C),^16^^,^^17^ whereas those using finer temporal resolutions (monthly mean temperatures) reported higher high-risk ranges (20-22°C).^21^ Additionally, a study conducted in a subtropical zone (Zhejiang)^20^ reported higher high-risk ranges (17-18°C), whereas 2 studies in a temperate zone (Shandong)^13^^,^^18^ identified lower ranges (around 12-13°C). Nationwide studies covering both climate zones tended to report broader high-risk ranges.^15^^-^^17^ Other patterns identified by nonlinear modeling approaches (increasing and decreasing trends) may reflect either substantial uncertainty at temperature extremes^22^^,^^25^^,^^26^ or constraints arising from analyses limited to narrow ranges of temperature (often due to the use of annual metrics).^13^^,^^20^

### Humidity

A total of 11 studies investigated the relationship with humidity, all of which used relative humidity as the primary metric (Table 2). Most focused on national or subnational regions in China, with 1 analyzing nationwide data from South Korea.^19^ Among 7 studies positing a nonlinear relationship, 5 identified increasing SFTS risk up to approximately 75% relative humidity. Of these 5, 2 reported a notable decline beyond this threshold, indicating a right-skewed reverse-U-shaped pattern.^16^^,^^21^ The remaining 3 reported a continued upward trend across the entire humidity range examined,^22^^,^^26^^,^^29^ although estimates at higher humidity levels were highly uncertain due to sparse data above 75%. Of 4 studies assuming a linear relationship, 3 found a positive association with SFTS incidence,^23^^,^^31^^,^^32^ whereas the study in South Korea reported a negative association (but not statistically significant at the 0.05 level) that was derived based on annual mean humidity data restricted to a narrow range of 66% to 69%.^19^

### Precipitation

Fourteen studies explored the relationship with precipitation, with 9 focusing on cumulative precipitation and 5 on mean values (Table 3). Studies applying nonlinear models to cumulative precipitation often reported right-skewed reverse-U-shaped associations that were characterized by a sharp increase in risk beyond a threshold (inflection point) followed by a sustained plateau and gradual decline.^16^^,^^34^ Although the overall pattern was similar, inflection points varied across studies, alongside substantial heterogeneities in precipitation metrics, including differences in spatial resolution (national vs subnational) and temporal scale (eg, cumulative precipitation during the driest quarter^17^). Such inconsistencies complicate cross-study comparisons, particularly between subtropical and temperate zones, where precipitation regimes differ markedly.^37^ Studies using mean precipitation measures lacked sufficient spatial and temporal granularity, limiting the ability to draw generalized conclusions.^13^^,^^18^^,^^23^^,^^24^^,^^31^

### Elevation

Among 9 studies examining the relationship with elevation (ie, the height above mean sea level), all but 1 (which assumed a linear relationship^14^) reported a reverse-U-shaped pattern (Table S5). Across these studies, low-to-moderate elevations were consistently associated with the highest SFTS risk, forming a right-skewed reverse-U-shaped pattern, although the absolute magnitude of elevation varied across studies. Specifically, studies in China typically reported high-risk ranges between 100 and 400 m, corresponding to hilly (<200 m) and low-mountain (200-500 m) zones based on their national topographic classification.^38^ In contrast, a nationwide study from Japan identified peak SFTS hospitalization risk at much lower elevations (9.8-29.7 m; classified as mid-elevation within that study), likely reflecting the overall lower elevation profile of the study regions, where the maximum elevation analyzed was 114 m.^25^

### Land cover

Twelve studies examined the relationship between land cover and SFTS occurrence (Table S6), with the majority (*n* = 9) accounting for elevation as a potential confounder, given that higher elevations generally correspond to mountainous areas. Metrics for land cover varied across studies: 3 studies used the normalized difference vegetation index (NDVI) (ranging from −1 to 1), and the remaining 9 used the percentage coverage of specific land types. Among the NDVI-based studies, 2 conducted in a temperate zone (Shandong) identified a reverse-U-shaped pattern, with SFTS risk peaking in moderately vegetated areas (NDVI range, 0.3-0.75) and declining in more densely vegetated areas.^13^^,^^34^ In contrast, a study conducted in a subtropical zone (Zhejiang) reported a monotonically decreasing trend, with the high-risk range spanning a broad NDVI range (0-0.75).^20^ Among studies using specific land types, heterogeneity in the categories considered (eg, forest, shrubland, grassland, cropland, farmland, water bodies) limited direct cross-study comparisons.

### Other environmental factors

The roles of sunshine duration, wind speed, and air pressure in SFTS outcomes have been explored in a limited number of studies, with no more than 6 examining each variable. Additionally, substantial heterogeneity in measurement approaches further complicates cross-study comparisons and synthesis (Tables S7-S9). Given these, the current evidence remains insufficient to draw definitive conclusions regarding their influence on SFTS outcomes.

## Discussion

Environmental factors shape the transmission dynamics of vector-borne diseases, determining when and where transmission occurs. This role is particularly pronounced in the case of SFTS, where transmission to humans arises from a multihost spillover system in which environmental conditions simultaneously affect tick dynamics, animal reservoir activity, and human exposure. To characterize these relationships, our systematic review of 2910 studies synthesizes evidence across multiple geographic settings, measurement scales, and analytical frameworks. Despite considerable heterogeneity in study designs, environmental factors—including temperature, humidity, precipitation, elevation, and land cover—consistently exhibited nonlinear associations with human SFTS occurrence, most commonly taking the form of reverse-U-shaped patterns. These findings reinforce the necessity of analytic frameworks that can accommodate nonlinear dynamics when inferring environmental effects that shape SFTS transmission risk. However, we found no quantitative assessment of how environmental factors influence SFTS occurrences in the dominant tick vector (*H. longicornis*) or animal reservoirs, despite such studies falling within our search strategy and thus expected to be captured. This absence reveals a critical blind spot, emphasizing the need for future research that integrates multihost surveillance beyond human outcomes to better elucidate how environmental drivers shape SFTSV ecology at the tick–animal–human interface.

These reverse-U-shaped relationships are best understood within a broader One Health perspective, where SFTS occurrence in humans serves as a downstream reflection of multilayered environmental effects cascading through transboundary interactions among ticks, animal reservoirs, and human populations.^39^ Such nonlinear patterns likely arise through at least 3 major pathways: (1) regulation of tick population dynamics, (2) modulation of viral circulation within vector populations, and (3) shift in human and animal reservoir behaviors that affect spillover risk. First, environmental conditions—particularly temperature and humidity—play a central role in shaping the population dynamics and behavior of *H. longicornis*. Although this species tolerates a broad thermal gradient,^40^ environmental extremes at either end disrupt its development, questing activity, and survival. High temperatures delay molting and suppress questing activity, with sharp increases in death when the temperature is above 40°C.^40^ Conversely, extreme cold elevates overwintering mortality rates and reduces egg hatchability, limiting population growth in the following season.^41^ Similarly, low humidity increases larval desiccation, whereas excessive humidity (>85%) impairs questing activity and heightens vulnerability to microbial infections.^26^

Second, environmental conditions also critically shape the mechanisms that sustain SFTSV circulation within tick populations. Accumulating evidence suggests that both horizontal (via co-feeding) and vertical (via transovarial pathways) transmission pathways play essential roles in maintaining viral persistence in tick populations,^42^ yet these mechanisms are highly sensitive to environmental stressors. Extreme temperature and humidity can impair tick survival, reproduction, and life cycle progression, thereby constraining vertical transmission through reduced oviposition or larval viability and limiting horizontal transmission by suppressing questing activity or increasing the mortality rate. Additionally, extreme precipitation events (eg, heavy rainfall or flooding) may physically displace ticks from their habitats or hosts,^43^ further destabilizing viral circulation within tick populations.

Lastly, environmental factors also influence human behavior and wildlife movements, changing the likelihood of tick exposure. Outdoor activities associated with higher tick-exposure risks (eg, farming, forestry, recreational activities such as hiking or camping)^44^ are more frequent under moderate weather conditions and decline during climatic extremes. The observed reverse-U-shaped relationship with land cover (ie, coverage of grassland) may similarly reflect such behavioral dynamics; although ticks often flourish in densely vegetated environments, limited human access in some of these may reduce spillover opportunities to humans.

However, translating these environmental associations into actionable control strategies requires an integrated perspective because SFTSV transmission reflects the interplay of multiple environmental conditions rather than any single factor acting in isolation. Endemic hotspots of SFTS often emerge where favorable ecological conditions converge, offsetting constraints imposed by individual drivers. For instance, Gangwon province in South Korea, despite experiencing the nation’s coldest winter temperatures (−4 to 0°C on average^45^), consistently reports the highest human incidence of SFTS.^46^ This paradoxical pattern likely stems from the region’s extensive mountainous terrain, which provides suitable habitats for ticks and wildlife reservoirs, enabling sustained spillover to humans even under otherwise limiting climatic conditions.

Beyond environmental drivers, human sociocultural practices also introduce an additional layer of complexity to SFTS transmission, leading to distinct epidemiologic patterns even under comparable environmental conditions. China and Japan exhibit similar seasonal trends in human SFTS incidence—peaking in early summer (May-June) before gradually declining into autumn; however, South Korea shows a distinct pattern, with cases increasing in early summer but peaking sharply in October before dropping to near zero by November (Figure S1). Several factors may contribute to this divergence (eg, differences in agricultural practices such as dual harvesting in some regions of China); however, the generally comparable environmental conditions across the 3 countries,^47^ which likely support similar tick ecology, suggest sociocultural behaviors in humans may play a key role. In South Korea, outdoor activities surge during Korean Thanksgiving (Chuseok) in late September and early October, when many people visit ancestral graves located in vegetation-rich areas. This seasonal increase in outdoor exposure,^48^ coupled with heightened agricultural activity (eg, wild food foraging^44^), likely elevates the risk of tick encounters, contributing to the delayed seasonal peak in case counts. Furthermore, sporadic human SFTSV infections have been linked to direct contact with infected livestock or companion animals, particularly through exposure to blood or body fluids, placing veterinarians and animal handlers at heightened risk.^49^

Although our systematic review shows that reverse-U-shaped relationships are consistently reported across environmental factors, generalizing key features (such as high-risk ranges) remains challenging due to methodological heterogeneity. Much of this variation is driven by differences in spatiotemporal resolution in both environmental exposures and SFTS outcomes. To compensate for low SFTS incidence (typically fewer than 10 cases per month at the regional level in South Korea and Japan^50^^,^^51^), studies often rely on coarser resolution to maintain statistical power. However, this introduces a critical trade-off, because such aggregations may obscure underlying environmental effects by artificially shifting or broadening estimated high-risk ranges. Indeed, studies using coarse spatiotemporal resolutions tend to report lower and more dispersed high-risk temperature ranges compared with more granular analyses. This likely reflects the smoothing of fluctuations under coarse aggregation, which can distort the apparent shape of environmental effects.

Methodological heterogeneity is further compounded by differing modeling frameworks, particularly regarding how lagged effects are handled. Given the likely time delay between environmental exposure and human SFTS occurrence, incorporating lag structures within the model frameworks can fundamentally reshape the estimated exposure-response relationship, underscoring the importance of applying approaches that explicitly incorporate such time lags (eg, distributed lag models). However, although 5 studies used models with lag structure,^21^^,^^23^^,^^28-30^ the impact of these methodological differences was not clearly evident in our review, likely reflecting the aforementioned limitation of coarse data resolution. When the aggregation interval exceeds the duration of underlying time lags, the resulting smoothing averages out time-dependent signals. This attenuation masks the comparative performance of different modeling approaches. Hence, robust inference would require high-resolution data alongside application of modeling frameworks capable of capturing these time-dependent dynamics.

Beyond improving data resolutions, future research should move toward refined analytical approaches capable of disentangling stage-specific environmental effects across the SFTS transmission cycle. Although prior studies have advanced understanding of key environmental correlates, most have relied on analytic frameworks that link environmental variables solely to human SFTS outcomes—1 end point of a transboundary transmission process. Such approaches collapse multifaceted environmental effects into overly simplified estimates, obscuring how specific environmental drivers operate at different stages of SFTS transmission, from shaping tick population dynamics and viral persistence within vectors to modulating transmission among animal reservoirs. This simplification also complicates the interpretation of estimated time lags between environmental exposure and SFTS outcomes, because lag structures are likely to be context specific and stage dependent.

In this context, mechanistic (mathematical) modeling grounded in the One Health framework^52^ offers a promising way forward. By explicitly incorporating environmental influences into the ecological dynamics of vectors and animal reservoirs, as well as the stage-specific transmission dynamics of SFTSV, these models can elucidate how environmental factors act at each stage of the transmission cycle across the tick–animal–human interface—an insight fundamentally inaccessible to conventional approaches that rely solely on simplified associations with human case counts. Moreover, incorporating human mobility into these frameworks can strengthen inference by partially addressing spatial misclassification inherent in residence-based incidence data, accounting for infections acquired outside of the residence region but recorded after they return and seek care in their home region. However, mechanistic models typically require assumptions on the underlying transmission dynamics, including the functional form of environmental effects, whereas statistical modeling and machine-learning approaches infer those associations directly from previously observed data. To reconcile these perspectives, the findings from this review bridge this gap by providing an empirical basis for informing plausible parameterization in mechanistic models, particularly in light of the nonlinear relationships identified across environmental factors.

In parallel, mechanistic modeling efforts must be supported by robust empirical data that capture the broader ecological context of SFTSV transmission. This requires surveillance systems that extend beyond human cases to systematically collect serological and genomic data across vectors, animal reservoirs, and human populations. Such multihost surveillance is essential for clarifying underlying patterns of host exposure, viral evolution, and interspecies transmission—core components of a One Health understanding of SFTS transmission dynamics. Furthermore, as the virus continues expanding its geographic range—demonstrated by the ongoing northward shift of SFTS endemic zones in Japan^53^—and as climate change and migratory bird movements increase the likelihood of long-distance tick dispersal,^54^^,^^55^ surveillance must also extend to regions not previously considered at risk. Regional monitoring will be critical for detecting emerging hotspots, tracking shifts in transmission dynamics (including changes in tick species composition or dominance^56^), and enhancing our understanding of how environmental and anthropogenic factors are shaping the evolving landscape of SFTSV transmission across the tick–animal–human interface.

Several limitations must be noted. First, a quantitative synthesis (eg, meta-analysis) of evidence on environmental effects on SFTS occurrence was not conducted in this review, because substantial heterogeneity in analytical frameworks across studies precluded pooling reported outcomes on a common quantitative scale (including exposure-response curves or regression coefficients). Second, for similar reasons, standardized schematic diagrams for individual environmental factors were not presented, because variation in exposure definitions and measurement scales across studies would imply cross-study comparability that is not methodologically warranted. Third, such heterogeneity across studies further limited detailed cross-study comparisons, including the identification of potential climate zone-specific patterns in environmental effects on SFTS occurrence. As additional studies with more comparable conditions become available, more robust quantitative syntheses and cross-study comparisons may be feasible in future work. Lastly, although analytical frameworks beyond linearity assumptions may influence reported environmental effects, these differences were not systematically evaluated, because this was beyond the primary scope of the review.

## Conclusion

Our systematic review suggests that environmental factors are associated with human SFTS occurrence, most often through nonlinear patterns. Beyond synthesizing existing evidence, we found that current evidence remains heavily focused on human outcomes while largely overlooking the multilayered environmental influences that shape SFTSV transmission across vectors and animal reservoirs. This imbalance represents a critical gap from a One Health perspective, obscuring the way in which environmental drivers shape transmission dynamics across the tick–animal–human interface and limiting our understanding of SFTSV circulation as an integrated ecological process. As SFTS continues to expand beyond historically endemic areas, future research must adopt a more integrative paradigm by applying mechanistic modeling approaches integrated within One Health surveillance to disentangle stage-specific environmental effects across the transmission cycle. In parallel, strengthened multihost surveillance and sustained scientific collaboration across regions and sectors will also be essential for enhancing inference on environmental effects and for supporting evidence-based control strategies to mitigate the growing transboundary burden of SFTS.

## Supplementary Material

Web_Material_mxag011

## Data Availability

No new data were generated while conducting this systematic review. All data synthesized in this study were derived from previously published articles included in the review and are available within this article and its supplementary materials.
